# *catena*-Poly[[[aqua­silver(I)]-μ-4,4′-bipyridine-κ^2^ *N*:*N*′] 4-amino­benzoate nitrate hydrate]

**DOI:** 10.1107/S1600536810005052

**Published:** 2010-02-13

**Authors:** Yong-Mei Zhang, Dong-Yan Hou, Guang Xin, Tie-Chun Li

**Affiliations:** a Department of Chemistry, Anshan Normal University, Anshan 114007, People’s Republic of China

## Abstract

In the structure of the title compound, 2[Ag(C_10_H_8_N_2_)(H_2_O)](C_7_H_6_NO_2_)(NO_3_)·H_2_O, the Ag^I^ atom is three-coordinated in a T-shaped configuration by two N atoms from two symmetry-related 4,4′-bipyridine (bipy) ligands at short distances and by one water O atom at a longer distance. Each bipy ligand bridges two neighbouring Ag^I^ atoms, forming a chain structure extending parallel to [101]. The complete 4-amino­benzoate anion, the nitrate anion and the uncoordinated water mol­ecule are located on mirror planes: together with the coordinated water mol­ecule, they form N—H⋯O, O—H⋯O and O—H⋯N hydrogen bonds, stabilizing the crystal structure.

## Related literature

For a related structure, see: Zhang *et al.* (2008 ▶).
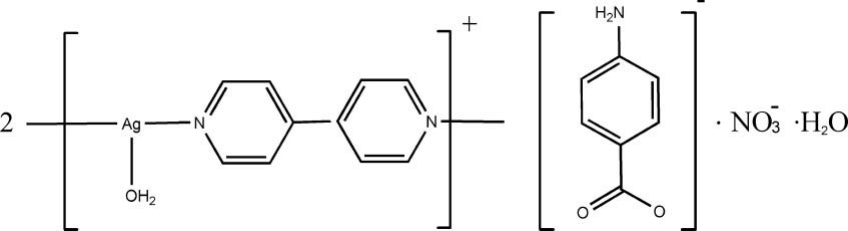


## Experimental

### 

#### Crystal data

2[Ag(C_10_H_8_N_2_)(H_2_O)](C_7_H_6_NO_2_)(NO_3_)·H_2_O
*M* *_r_* = 780.29
Monoclinic, 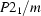

*a* = 8.2595 (4) Å
*b* = 17.3531 (8) Å
*c* = 9.9267 (4) Å
β = 103.231 (1)°
*V* = 1385.01 (11) Å^3^
*Z* = 2
Mo *K*α radiation
μ = 1.48 mm^−1^
*T* = 293 K
0.24 × 0.21 × 0.17 mm

#### Data collection

Bruker APEX CCD area-detector diffractometer
Absorption correction: multi-scan (*SADABS*; Sheldrick, 1996 ▶) *T*_min_ = 0.54, *T*_max_ = 0.83
7782 measured reflections
2847 independent reflections
2390 reflections with *I* > 2σ(*I*)
*R*_int_ = 0.024

#### Refinement

*R*[*F* ^2^ > 2σ(*F* ^2^)] = 0.023
*wR*(*F* ^2^) = 0.059
*S* = 1.07
2847 reflections
230 parameters
8 restraints
H atoms treated by a mixture of independent and constrained refinement
Δρ_max_ = 0.34 e Å^−3^
Δρ_min_ = −0.38 e Å^−3^

### 

Data collection: *SMART* (Bruker, 1998 ▶); cell refinement: *SAINT* (Bruker, 1998 ▶); data reduction: *SAINT*; program(s) used to solve structure: *SHELXS97* (Sheldrick, 2008 ▶); program(s) used to refine structure: *SHELXL97* (Sheldrick, 2008 ▶); molecular graphics: *SHELXTL* (Sheldrick, 2008 ▶); software used to prepare material for publication: *SHELXTL*.

## Supplementary Material

Crystal structure: contains datablocks global, I. DOI: 10.1107/S1600536810005052/wm2305sup1.cif

Structure factors: contains datablocks I. DOI: 10.1107/S1600536810005052/wm2305Isup2.hkl

Additional supplementary materials:  crystallographic information; 3D view; checkCIF report

## Figures and Tables

**Table:** 

Ag1—N3^i^	2.139 (2)
Ag1—N2	2.1444 (19)
Ag1—O1*W*^ii^	2.6799 (17)

**Table:** 

N3^i^—Ag1—N2	172.70 (7)
N3^i^—Ag1—O1*W*^ii^	92.88 (6)
N2—Ag1—O1*W*^ii^	90.84 (6)

Symmetry codes: (i) 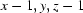; (ii) 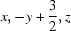.

**Table 2:** Hydrogen-bond geometry (Å, °)

*D*—H⋯*A*	*D*—H	H⋯*A*	*D*⋯*A*	*D*—H⋯*A*
N1—H1*A*⋯O2*W*^iii^	0.85 (4)	2.34 (2)	3.160 (5)	163
N1—H1*B*⋯O2^iv^	0.85 (4)	2.10 (4)	2.932 (4)	167
O1*W*—H*W*11⋯O1	0.85 (3)	1.91 (3)	2.747 (2)	172 (3)
O1*W*—H*W*12⋯O4	0.83 (3)	2.15 (3)	2.927 (3)	154 (3)
O2*W*—H*W*21⋯O1	0.85 (3)	2.14 (3)	2.979 (4)	169 (3)
O2*W*—H*W*22⋯O5^v^	0.86 (3)	2.30 (2)	3.084 (3)	151
O2*W*—H*W*22⋯O5^vi^	0.86 (3)	2.30 (2)	3.084 (3)	151
O2*W*—H*W*22⋯N4^v^	0.86 (3)	2.63 (3)	3.476 (4)	167

Symmetry codes: (iii) 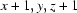; (iv) 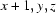; (v) 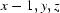; (vi) 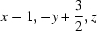.

**Fig. 1.**
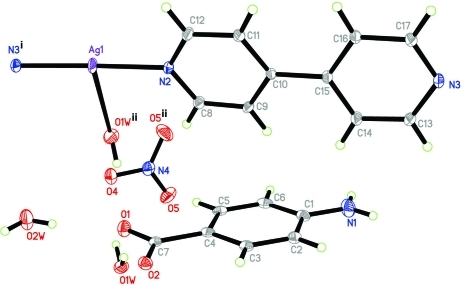
The structure of (I), showing the atomic numbering scheme. Displacement ellipsoids are drawn at the 30% probability level. [Symmetry codes: (i) x-1, y, z-1; (ii) x, 1.5-y, z]

## supplementary crystallographic information

### Comment

Silver coordination polymers have received intense interests because of their interesting structural features and potential applications (Zhang *et al.*, 2008). We report here the synthesis and structure of the title compound, [Ag_2_(C_10_H_8_N_2_)_2_(H_2_O)_2_].(C_7_H_6_NO_2_).NO_3_.H_2_O, (I).

In the crystal structure of compound (I), the Ag^I^ atom is three-coordinated by two nitrogen atoms from two symmetry-related bipy (bipy = 4,4'-bipyridine) ligands and one water water molecule in a T-shaped coordination configuration (Fig. 1). Each bipy ligand bridges two neighboring Ag^I^ atoms to form a chain structure along [101]. Further, *L* anions (*L* = 4-aminobenzoate), the nitrate anion, and the uncoordinated water molecule form N—H···O and O—H···O hydrogen bonds, stabilizing the structure of (I).

### Experimental

A mixture of AgNO_3_ (1 mmol), NaOH (0.040 g, 1 mmol) and 4-aminobenzoic acid (1 mmol) in water (15 ml) was stirred for 10 min at room temperature. Then 4,4'-bipyridine (1 mmol) was added to the solution with stirring for 30 min and a white precipitate was obtained. The precipitate was dissolved by dropwise addition of ammonia (5 M). Single crystals were obtained by slow evaporation of the solution at room temperature.

### Refinement

All H atoms on C atoms were positioned geometrically (C—H = 0.93 Å) and refined as riding, with U_iso_(H) = 1.2U_eq_(carrier). The amino and water H atoms were located in a difference Fourier map, and were refined with a distance restraints of N—H = O—H = 0.85 Å.

### Crystal data

**Table:**

2[Ag(C_10_H_8_N_2_)(H_2_O)](C_7_H_6_NO_2_)(NO_3_)·H_2_O	*F*(000) = 780
*M**_r_* = 780.29	*D*_x_ = 1.871 Mg m^−^^3^
Monoclinic, *P*2_1_/*m*	Mo *K*α radiation, λ = 0.71073 Å
Hall symbol: -P 2yb	Cell parameters from 2847 reflections
*a* = 8.2595 (4) Å	θ = 2.1–26.1°
*b* = 17.3531 (8) Å	µ = 1.48 mm^−^^1^
*c* = 9.9267 (4) Å	*T* = 293 K
β = 103.231 (1)°	Block, colourless
*V* = 1385.01 (11) Å^3^	0.24 × 0.21 × 0.17 mm
*Z* = 2	

### Data collection

**Table:**

Bruker APEX CCD area-detector diffractometer	2847 independent reflections
Radiation source: fine-focus sealed tube	2390 reflections with *I* > 2σ(*I*)
graphite	*R*_int_ = 0.024
φ and ω scans	θ_max_ = 26.1°, θ_min_ = 2.1°
Absorption correction: multi-scan (*SADABS*; Sheldrick, 1996)	*h* = −10→7
*T*_min_ = 0.54, *T*_max_ = 0.83	*k* = −21→17
7782 measured reflections	*l* = −12→12

### Refinement

**Table:**

Refinement on *F*^2^	Primary atom site location: structure-invariant direct methods
Least-squares matrix: full	Secondary atom site location: difference Fourier map
*R*[*F*^2^ > 2σ(*F*^2^)] = 0.023	Hydrogen site location: inferred from neighbouring sites
*wR*(*F*^2^) = 0.059	H atoms treated by a mixture of independent and constrained refinement
*S* = 1.07	*w* = 1/[σ^2^(*F*_o_^2^) + (0.0284*P*)^2^ + 0.2523*P*] where *P* = (*F*_o_^2^ + 2*F*_c_^2^)/3
2847 reflections	(Δ/σ)_max_ = 0.003
230 parameters	Δρ_max_ = 0.34 e Å^−^^3^
8 restraints	Δρ_min_ = −0.38 e Å^−^^3^

### Special details

**Table:**

Geometry. All esds (except the esd in the dihedral angle between two l.s. planes) are estimated using the full covariance matrix. The cell esds are taken into account individually in the estimation of esds in distances, angles and torsion angles; correlations between esds in cell parameters are only used when they are defined by crystal symmetry. An approximate (isotropic) treatment of cell esds is used for estimating esds involving l.s. planes.
Refinement. Refinement of *F*^2^ against ALL reflections. The weighted *R*-factor wR and goodness of fit *S* are based on *F*^2^, conventional *R*-factors *R* are based on *F*, with *F* set to zero for negative *F*^2^. The threshold expression of *F*^2^ > σ(*F*^2^) is used only for calculating *R*-factors(gt) etc. and is not relevant to the choice of reflections for refinement. *R*-factors based on *F*^2^ are statistically about twice as large as those based on *F*, and *R*- factors based on ALL data will be even larger.

### Fractional atomic coordinates and isotropic or equivalent isotropic displacement parameters (Å^2^)

**Table:**

	*x*	*y*	*z*	*U*_iso_*/*U*_eq_	
C1	0.9046 (4)	0.7500	0.9999 (4)	0.0234 (8)	
C2	0.7795 (4)	0.7500	1.0736 (4)	0.0216 (7)	
H2	0.8082	0.7500	1.1698	0.026*	
C3	0.6139 (4)	0.7500	1.0056 (4)	0.0201 (7)	
H3	0.5331	0.7500	1.0573	0.024*	
C4	0.5645 (4)	0.7500	0.8621 (3)	0.0198 (7)	
C5	0.6902 (4)	0.7500	0.7886 (4)	0.0215 (7)	
H5	0.6610	0.7500	0.6924	0.026*	
C6	0.8556 (4)	0.7500	0.8547 (4)	0.0240 (8)	
H6	0.9361	0.7500	0.8027	0.029*	
C7	0.3820 (4)	0.7500	0.7909 (3)	0.0198 (7)	
C8	0.6051 (3)	0.95341 (14)	0.7979 (2)	0.0211 (5)	
H8	0.5907	0.9099	0.7417	0.025*	
C9	0.7206 (3)	0.95076 (14)	0.9219 (2)	0.0203 (5)	
H9	0.7822	0.9061	0.9477	0.024*	
C10	0.7458 (3)	1.01485 (13)	1.0091 (2)	0.0156 (5)	
C11	0.6492 (3)	1.07948 (14)	0.9627 (2)	0.0204 (5)	
H11	0.6611	1.1238	1.0166	0.025*	
C12	0.5365 (3)	1.07780 (14)	0.8371 (2)	0.0219 (5)	
H12	0.4739	1.1218	0.8082	0.026*	
C13	1.0736 (3)	0.94885 (15)	1.3175 (2)	0.0235 (5)	
H13	1.1264	0.9031	1.3512	0.028*	
C14	0.9522 (3)	0.94648 (14)	1.1975 (2)	0.0238 (5)	
H14	0.9241	0.8998	1.1523	0.029*	
C15	0.8700 (3)	1.01438 (13)	1.1424 (2)	0.0172 (5)	
C16	0.9155 (3)	1.08119 (14)	1.2193 (2)	0.0210 (5)	
H16	0.8627	1.1276	1.1897	0.025*	
C17	1.0384 (3)	1.07876 (14)	1.3392 (2)	0.0231 (5)	
H17	1.0664	1.1243	1.3883	0.028*	
N1	1.0689 (4)	0.7500	1.0676 (4)	0.0366 (8)	
N2	0.5122 (2)	1.01603 (11)	0.7543 (2)	0.0180 (4)	
N3	1.1201 (2)	1.01423 (11)	1.3889 (2)	0.0206 (4)	
N4	0.6731 (4)	0.7500	0.3860 (3)	0.0293 (7)	
O1	0.3438 (3)	0.7500	0.6599 (3)	0.0305 (6)	
O2	0.2780 (3)	0.7500	0.8655 (2)	0.0234 (5)	
O1W	0.4203 (2)	0.62838 (10)	0.50869 (19)	0.0327 (4)	
O2W	0.0951 (4)	0.7500	0.3901 (3)	0.0499 (8)	
O4	0.5190 (3)	0.7500	0.3396 (3)	0.0365 (7)	
O5	0.7501 (3)	0.68814 (12)	0.4064 (2)	0.0511 (6)	
Ag1	0.32721 (2)	1.014048 (12)	0.563502 (18)	0.02456 (8)	
H1A	1.099 (5)	0.7500	1.155 (2)	0.046 (14)*	
H1B	1.143 (4)	0.7500	1.021 (4)	0.044 (13)*	
HW11	0.400 (4)	0.6633 (16)	0.562 (3)	0.065*	
HW12	0.448 (4)	0.6508 (18)	0.443 (2)	0.065*	
HW21	0.154 (4)	0.7500	0.472 (3)	0.065*	
HW22	−0.004 (3)	0.7500	0.404 (4)	0.065*	

### Atomic displacement parameters (Å^2^)

**Table:**

	*U*^11^	*U*^22^	*U*^33^	*U*^12^	*U*^13^	*U*^23^
C1	0.0206 (18)	0.0152 (18)	0.035 (2)	0.000	0.0080 (15)	0.000
C2	0.0238 (18)	0.0190 (18)	0.0225 (18)	0.000	0.0064 (14)	0.000
C3	0.0230 (18)	0.0137 (17)	0.0254 (18)	0.000	0.0093 (14)	0.000
C4	0.0234 (18)	0.0109 (17)	0.0267 (19)	0.000	0.0089 (15)	0.000
C5	0.032 (2)	0.0118 (17)	0.0234 (18)	0.000	0.0116 (15)	0.000
C6	0.0270 (19)	0.0154 (18)	0.034 (2)	0.000	0.0171 (16)	0.000
C7	0.0244 (18)	0.0092 (16)	0.0246 (19)	0.000	0.0033 (15)	0.000
C8	0.0243 (13)	0.0189 (13)	0.0175 (12)	0.0005 (10)	−0.0003 (10)	−0.0022 (10)
C9	0.0244 (13)	0.0145 (12)	0.0187 (12)	0.0019 (10)	−0.0022 (10)	0.0011 (10)
C10	0.0140 (11)	0.0174 (12)	0.0146 (11)	−0.0019 (9)	0.0012 (9)	0.0016 (9)
C11	0.0233 (13)	0.0156 (12)	0.0194 (12)	0.0004 (9)	−0.0013 (10)	−0.0031 (9)
C12	0.0222 (12)	0.0192 (13)	0.0208 (12)	0.0054 (10)	−0.0027 (10)	0.0010 (10)
C13	0.0256 (13)	0.0180 (13)	0.0228 (13)	0.0017 (10)	−0.0030 (10)	0.0021 (10)
C14	0.0274 (13)	0.0177 (13)	0.0211 (12)	0.0012 (10)	−0.0051 (10)	−0.0023 (10)
C15	0.0140 (11)	0.0198 (12)	0.0166 (12)	−0.0008 (9)	0.0007 (9)	0.0013 (9)
C16	0.0205 (12)	0.0180 (13)	0.0207 (12)	0.0017 (10)	−0.0034 (10)	−0.0013 (10)
C17	0.0256 (13)	0.0179 (13)	0.0217 (13)	−0.0005 (10)	−0.0031 (10)	−0.0025 (10)
N1	0.0192 (17)	0.051 (2)	0.039 (2)	0.000	0.0064 (17)	0.000
N2	0.0179 (10)	0.0195 (10)	0.0145 (10)	−0.0001 (8)	−0.0007 (8)	0.0017 (8)
N3	0.0199 (10)	0.0223 (11)	0.0161 (10)	−0.0004 (9)	−0.0029 (8)	−0.0005 (8)
N4	0.0349 (19)	0.035 (2)	0.0175 (15)	0.000	0.0045 (14)	0.000
O1	0.0278 (14)	0.0388 (16)	0.0232 (14)	0.000	0.0024 (11)	0.000
O2	0.0211 (13)	0.0210 (13)	0.0287 (14)	0.000	0.0071 (11)	0.000
O1W	0.0407 (12)	0.0278 (11)	0.0273 (11)	−0.0025 (9)	0.0030 (9)	0.0029 (8)
O2W	0.0302 (16)	0.062 (2)	0.051 (2)	0.000	−0.0047 (14)	0.000
O4	0.0275 (15)	0.0436 (17)	0.0352 (16)	0.000	0.0005 (12)	0.000
O5	0.0491 (13)	0.0414 (14)	0.0585 (14)	0.0149 (11)	0.0034 (11)	0.0173 (11)
Ag1	0.02136 (12)	0.03052 (13)	0.01619 (11)	0.00025 (8)	−0.00732 (8)	−0.00028 (8)

### Geometric parameters (Å, °)

**Table:**

Ag1—N3^i^	2.139 (2)	C17—H17	0.9300
Ag1—N2	2.1444 (19)	O1—C7	1.266 (4)
Ag1—O1W^ii^	2.6799 (17)	O2—C7	1.255 (4)
N2—C12	1.337 (3)	N1—C1	1.369 (4)
N2—C8	1.344 (3)	N1—H1A	0.85 (4)
N3—C17	1.342 (3)	N1—H1B	0.85 (4)
N3—C13	1.346 (3)	C1—C2	1.396 (5)
N3—Ag1^iii^	2.139 (2)	C1—C6	1.405 (5)
C8—C9	1.374 (3)	C2—C3	1.380 (4)
C8—H8	0.9300	C2—H2	0.9300
C9—C10	1.396 (3)	C3—C4	1.389 (5)
C9—H9	0.9300	C3—H3	0.9300
C10—C11	1.392 (3)	C4—C5	1.399 (4)
C10—C15	1.477 (3)	C4—C7	1.511 (4)
C11—C12	1.375 (3)	C5—C6	1.373 (5)
C11—H11	0.9300	C5—H5	0.9300
C12—H12	0.9300	C6—H6	0.9300
C13—C14	1.371 (3)	O4—N4	1.251 (4)
C13—H13	0.9300	O5—N4	1.240 (2)
C14—C15	1.407 (3)	N4—O5^ii^	1.240 (2)
C14—H14	0.9300	O1W—HW11	0.85 (2)
C15—C16	1.392 (3)	O1W—HW12	0.85 (2)
C16—C17	1.377 (3)	O2W—HW21	0.85 (2)
C16—H16	0.9300	O2W—HW22	0.85 (2)
			
N3^i^—Ag1—N2	172.70 (7)	C17—C16—H16	120.0
N3^i^—Ag1—O1W^ii^	92.88 (6)	C15—C16—H16	120.0
N2—Ag1—O1W^ii^	90.84 (6)	N3—C17—C16	123.6 (2)
C12—N2—C8	117.1 (2)	N3—C17—H17	118.2
C12—N2—Ag1	122.13 (15)	C16—C17—H17	118.2
C8—N2—Ag1	120.71 (16)	C1—N1—H1A	122 (3)
C17—N3—C13	116.8 (2)	C1—N1—H1B	119 (3)
C17—N3—Ag1^iii^	122.74 (16)	H1A—N1—H1B	119 (3)
C13—N3—Ag1^iii^	120.27 (16)	N1—C1—C2	120.8 (3)
N2—C8—C9	122.9 (2)	N1—C1—C6	121.6 (3)
N2—C8—H8	118.5	C2—C1—C6	117.6 (3)
C9—C8—H8	118.5	C3—C2—C1	120.9 (3)
C8—C9—C10	120.2 (2)	C3—C2—H2	119.5
C8—C9—H9	119.9	C1—C2—H2	119.5
C10—C9—H9	119.9	C2—C3—C4	121.8 (3)
C11—C10—C9	116.4 (2)	C2—C3—H3	119.1
C11—C10—C15	121.9 (2)	C4—C3—H3	119.1
C9—C10—C15	121.7 (2)	C3—C4—C5	117.1 (3)
C12—C11—C10	120.1 (2)	C3—C4—C7	120.4 (3)
C12—C11—H11	120.0	C5—C4—C7	122.5 (3)
C10—C11—H11	120.0	C6—C5—C4	121.8 (3)
N2—C12—C11	123.3 (2)	C6—C5—H5	119.1
N2—C12—H12	118.3	C4—C5—H5	119.1
C11—C12—H12	118.3	C5—C6—C1	120.8 (3)
N3—C13—C14	123.1 (2)	C5—C6—H6	119.6
N3—C13—H13	118.4	C1—C6—H6	119.6
C14—C13—H13	118.4	O2—C7—O1	124.2 (3)
C13—C14—C15	120.3 (2)	O2—C7—C4	117.9 (3)
C13—C14—H14	119.8	O1—C7—C4	117.9 (3)
C15—C14—H14	119.8	O5—N4—O5^ii^	119.9 (3)
C16—C15—C14	116.1 (2)	O5—N4—O4	120.02 (16)
C16—C15—C10	122.1 (2)	O5^ii^—N4—O4	120.02 (16)
C14—C15—C10	121.8 (2)	HW11—O1W—HW12	107 (3)
C17—C16—C15	120.1 (2)	HW21—O2W—HW22	102 (3)
			
N3^i^—Ag1—N2—C12	−61.6 (6)	C9—C10—C15—C14	−9.2 (4)
N3^i^—Ag1—N2—C8	116.0 (6)	C14—C15—C16—C17	2.1 (4)
C12—N2—C8—C9	0.4 (3)	C10—C15—C16—C17	−176.4 (2)
Ag1—N2—C8—C9	−177.37 (18)	C13—N3—C17—C16	−1.8 (4)
N2—C8—C9—C10	0.0 (4)	Ag1^iii^—N3—C17—C16	173.52 (19)
C8—C9—C10—C11	−0.3 (3)	C15—C16—C17—N3	−0.1 (4)
C8—C9—C10—C15	−179.3 (2)	N1—C1—C2—C3	180.000 (2)
C9—C10—C11—C12	0.1 (3)	C6—C1—C2—C3	0.000 (2)
C15—C10—C11—C12	179.2 (2)	C1—C2—C3—C4	0.000 (2)
C8—N2—C12—C11	−0.6 (4)	C2—C3—C4—C5	0.000 (2)
Ag1—N2—C12—C11	177.15 (18)	C2—C3—C4—C7	180.000 (2)
C10—C11—C12—N2	0.3 (4)	C3—C4—C5—C6	0.000 (2)
C17—N3—C13—C14	1.6 (4)	C7—C4—C5—C6	180.000 (1)
Ag1^iii^—N3—C13—C14	−173.80 (19)	C4—C5—C6—C1	0.000 (2)
N3—C13—C14—C15	0.4 (4)	N1—C1—C6—C5	180.000 (2)
C13—C14—C15—C16	−2.3 (4)	C2—C1—C6—C5	0.000 (2)
C13—C14—C15—C10	176.3 (2)	C3—C4—C7—O2	0.000 (1)
C11—C10—C15—C16	−9.7 (4)	C5—C4—C7—O2	180.000 (1)
C9—C10—C15—C16	169.3 (2)	C3—C4—C7—O1	180.000 (1)
C11—C10—C15—C14	171.8 (2)	C5—C4—C7—O1	0.000 (1)

Symmetry codes: (i) *x*−1, *y*, *z*−1; (ii) *x*, −*y*+3/2, *z*; (iii) *x*+1, *y*, *z*+1.

### Hydrogen-bond geometry (Å, °)

**Table:**

*D*—H···*A*	*D*—H	H···*A*	*D*···*A*	*D*—H···*A*
N1—H1A···O2W^iii^	0.85 (4)	2.34 (2)	3.160 (5)	163
N1—H1B···O2^iv^	0.85 (4)	2.10 (4)	2.932 (4)	167
O1W—HW11···O1	0.85 (3)	1.91 (3)	2.747 (2)	172 (3)
O1W—HW12···O4	0.83 (3)	2.15 (3)	2.927 (3)	154 (3)
O2W—HW21···O1	0.85 (3)	2.14 (3)	2.979 (4)	169 (3)
O2W—HW22···O5^v^	0.86 (3)	2.30 (2)	3.084 (3)	151
O2W—HW22···O5^vi^	0.86 (3)	2.30 (2)	3.084 (3)	151
O2W—HW22···N4^v^	0.86 (3)	2.63 (3)	3.476 (4)	167

Symmetry codes: (iii) *x*+1, *y*, *z*+1; (iv) *x*+1, *y*, *z*; (v) *x*−1, *y*, *z*; (vi) *x*−1, −*y*+3/2, *z*.
